# Degenerative findings on MRI of the cervical spine: an inter- and intra-rater reliability study

**DOI:** 10.1186/s12998-018-0210-2

**Published:** 2018-10-16

**Authors:** Line Thorndal Moll, Morten Wasmod Kindt, Christina Malmose Stapelfeldt, Tue Secher Jensen

**Affiliations:** 1 DEFACTUM, Central Denmark Region, P.P. Oerums Gade 11, bygn. 1B, DK-8000 Aarhus C, Denmark; 2 Section of Clinical Social Medicine and Rehabilitation, Department of Public Health, Aarhus University, P.P. Oerums Gade 9-11, bygn. 1B, DK-8000 Aarhus C, Denmark; 3 Spine Centre, Diagnostic Centre, University Research Clinic for Innovative Patient Pathways, Silkeborg Regional Hospital, Falkevej 1-3, DK-8600 Silkeborg, Denmark; 4 Department for Diagnostic Imaging, Diagnostic Centre, University Research Clinic for Innovative Patient Pathways, Silkeborg Regional Hospital, Falkevej 1-3, DK-8600 Silkeborg, Denmark; 5 Nordic Institute of Chiropractic and Clinical Biomechanics, University of Southern Denmark, Campusvej 55, DK-5230 Odense M, Denmark

**Keywords:** Magnetic resonance imaging, Reliability, Cervical spine, Degenerative, Classification, MRI, Agreement

## Abstract

**Background:**

Knowledge about the assessment reliability of common cervical spine changes is a prerequisite for precise and consistent communication about Magnetic Resonance Imaging (MRI) findings. The purpose of this study was to determine the inter- and intra-rater reliability of degenerative findings when assessing cervical spine MRI.

**Methods:**

Fifty cervical spine MRIs from subjects with neck pain were used. A radiologist, a chiropractor and a second-year resident of rheumatology independently assessed kyphosis, disc height, disc contour, vertebral endplate signal changes, spinal canal stenosis, neural foraminal stenosis, and osteoarthritis of the uncovertebral and zygapophyseal joints. An evaluation manual was composed containing classifications and illustrative examples, and ten of the MRIs were evaluated twice followed by consensus meetings to refine the classifications. Next, the three readers independently assessed the full sample. Reliability measures were reported using prevalence estimates and unweighted kappa (Κ) statistics.

**Results:**

The overall inter-rater reliability was substantial (Κ ≥ 0.61) for the majority of variables and moderate only for zygapophyseal osteoarthritis (Κ = 0.56). Intra-rater reliability estimates were higher for all findings.

**Conclusions:**

The present classifications for some of the most common cervical degenerative findings yielded mainly substantial inter-rater reliability estimates and substantial to almost perfect intra-rater reliability estimates. .

**Trial registration:**

Regional Data Protection Agency (J.no. 1–16–02-86-16). The letter of exemption from the Regional Ethical Committee is available from the author on request (case no. 86 / 2017).

**Electronic supplementary material:**

The online version of this article (10.1186/s12998-018-0210-2) contains supplementary material, which is available to authorized users.

## Background

Although not recommended as routine imaging in neck pain [1, 2], the number of cervical MRIs has increased by 18% compared to a 4.5% increase in neck pain prevalence over recent years in Denmark [2–4]. While patients believe in MRI to unveil the true cause of their pain [5], health care professionals appreciate the advantages of MRI compared with other modalities of diagnostic imaging. The non-invasiveness, absence of radiation exposure and the capacity to discriminate soft tissue changes are all highly valued in the field of musculoskeletal imaging.

When communicating MRI findings, the importance of consistency and precision remains unaltered. Both for academic and clinical purposes, a prerequisite for such consistency and precision is reliability in MRI assessments. Reliability is defined as “the extent to which scores for patients who have not changed are the same for repeated measurement under several conditions” [6]. In the case of MRI, this means that while the images do not change, reliability reflects whether the image interpretation remains the same when assessed by different raters (inter-rater reliability) or by the same rater at different times (intra-rater reliability).

Previous reliability studies on cervical spine MRI have found moderate to almost perfect inter-rater reliability in the assessments of disc-related parameters (kappa (Κ) 0.44[7], Κ 0.43–0.65 [8] and Κ 0.73–0.83 [9]). Almost perfect reliability has been reported for assessments of neural foraminal stenosis (Κ > 0.9 [10]), fair reliability for facet joint arthrosis (Κ 0.23–0.38 [11]), and moderate to substantial reliability for spinal canal stenosis (Κ 0.55–0.72 [11]). Most studies have focused on only one or a few degenerative variables [7–13] and compared readers with similar educational backgrounds and levels of experience [7–10, 12–14].

To our knowledge, only one reliability study on cervical spine MRI has covered a broad range of common degenerative findings [14] for which reason, further studies are needed.

### Objective

To determine the inter- and intra-rater assessment reliability of degenerative findings (kyphosis, disc height, disc contour, vertebral endplate signal changes, spinal canal stenosis, neural foraminal stenosis, uncovertebral osteoarthritis and zygapophyseal osteoarthritis) on MRI of the cervical spine.

## Methods

### Subjects

**Fig. 1:**
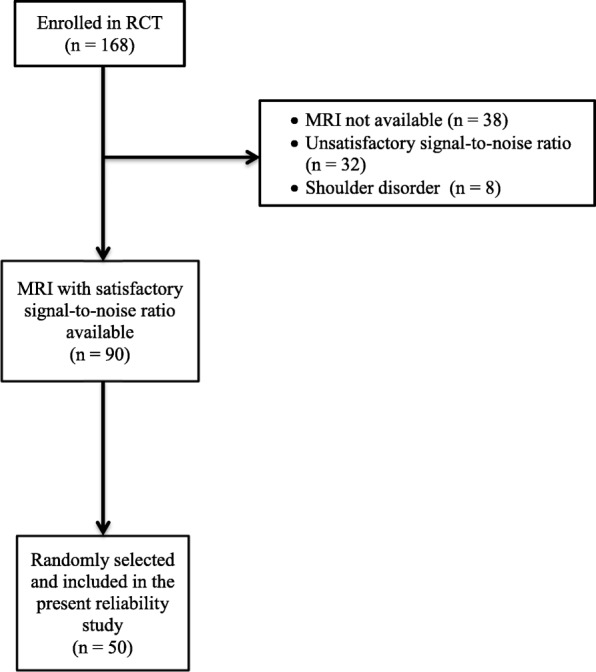
Flowchart

Fifty MRIs of the cervical spine were chosen from among subjects previously enrolled in a randomized controlled trial (RCT) [15]. Subjects for the RCT were recruited from primary health care professionals (physiotherapists, chiropractors and general practitioners (GPs)). If subjects fulfilled the inclusion criteria (age 18–60 years, part-time or full-time sick leave for 4–16 weeks owing to neck pain or shoulder pain, and fluency in Danish), their GPs referred them to The Spine Centre, Silkeborg Regional Hospital, Denmark. For the current study, the predefined inclusion criterion was the availability of a cervical spine MRI with a satisfactory signal-to-noise ratio. After assessment by the most experienced reader, 32 MRIs were excluded based on unsatisfactory signal-to-noise ratio. By choosing every second MRI among those remaining, 50 MRIs were selected for the current study. A study flow-chart is seen in Fig. 1.

### Data collection - images

The MRIs were provided from five different hospitals collaborating with The Spine Centre. The majority of the images were obtained using a 1.5 T field strength. All MRIs comprised sagittal T1-weighted and T2-weighted sequences, while an axial T2 sequence was available for 94% and oblique T2 sequences were available for 82% of the images.

### Data collection – readers

The three readers (Readers A, B and C) all assessed the images independently over a time frame of 5–8 weeks. Reader A was a second-year resident of rheumatology with no previous formal education in MRI assessment. She had 9 years of postgraduate clinical experience including assessment of spinal MRI for clinical purposes. Reader B was an experienced radiologist having worked with musculoskeletal MRI for 25 years, mostly on a daily basis. Reader C was a chiropractor who had completed a 1-year fulltime internship in spinal MRI in a radiology department. He had another 10 years of clinical and academic experience with spinal MRI. Prior to the study, Reader B taught Reader A assessment of cervical spine MRI for 2 h. Following this two-hour session, Reader A completed 50 clinical narrative reports of cervical spine MRIs from patients with neck pain with or without radiculopathy. These were not part of the current study. The reports were corrected if necessary and approved by Reader B.

For the intra-rater reliability assessment, Reader A assessed all the images twice. The second assessment took place after 6 weeks to prevent recollection of the first assessments.

### Evaluation manual, piloting and workstations

Based on the literature [10–14, 16–24], an evaluation manual with written and visual classifications of the findings was made by Reader A, adjusted and approved by Readers B and C. Next, 10 MRIs from the study sample were evaluated twice followed by consensus meetings. This piloting served the purpose of refining both the classifications in the evaluation manual and the practice of the readers. All images were de-identified, leaving the readers blinded to demographic and clinical data as well as previous assessments. The images were assessed on radiological work stations using Vitrea Core (version 1.0.0.404, Vital Images Inc.).

### Variables

Classifications for common and degenerative MRI findings were developed based primarily on the existing literature [10–13, 16–19, 23–26] and on experiences from the piloting. An effort was made to create definitions that were as simple as possible [14], assuming that simplicity is essential for clinical applicability. The most common degenerative findings were chosen, including kyphosis and vertebral endplate signal changes; all are routinely considered by radiologists assessing cervical spine MRIs at Silkeborg Regional Hospital. All the classifications yielded categorical (but not ordinal) data. The complete list of variables is presented in Table 1. Except for kyphosis, these findings were assessed for each of the six cervical disc levels (level C2/C3 to C7/T1). Furthermore, the neural foramina, uncovertebral and zygapophyseal joints were assessed separately on the left and right hand side. The evaluation manual is available in Additional file 1.

**Table 1:** MRI findings and corresponding classifications

MRI finding	Category	Description
Kyphosis	0	Normal or straightened lordosis
	1	Kyphosis
Disc height	0	Normal
	1	Reduced height
Disc contour	0	Normal
	1	Bulge or protrusion
	2	Extrusion
Spinal canal stenosis	0	Normal
	1	> 50% obliteration of CSF, no cord deformity
	2	> 50% obliteration of CSF with cord deformity but no signal change
	3	> 50% obliteration of CSF with cord deformity and signal change
Vertebral endplate signal change	0	Normal
	1	Type 1
	2	Type 2
	3	Type 3
	4	Mixed type 1 and 2
Uncovertebral osteoarthritis	0	Normal
	1	Definite osteoarthritis
Zygapophyseal osteoarthritis	0	Normal
	1	Definite osteoarthritis
Neural foraminal stenosis	0	Normal or < 50% fat obliteration
	1	≥ 50% fat obliteration with or without morphological changes of the nerve root

*CSF* cerebrospinal fluid

### Data entry and statistical analysis

All three readers independently entered and stored data using Epidata (Version 3.1., The EpiData Association, Odense, Denmark, 2003–2004). If assessment of a certain finding was not possible due to the available sequences, the particular finding was allotted the value ‘9’ representing ‘missing’.

In accordance with the recommendations for reliability studies [27], 50 MRIs were included in the current study. Prior to the kappa (Κ) calculations, all readers’ prevalence assessments were calculated, one variable at a time. This tabulation of data offered the opportunity of 1) assessing the sample homogeneity and 2) identifying any possible systematic differences between the readers; as both can affect the Κ estimates [27, 28]. Tabulation thus allowed for a clearer impression of agreement and possible misclassification than offered by the Κ value alone. Tabulation also provided estimates for observed agreement (OA) and agreement by chance (AC) for the pairwise analyses. For the overall three-reader analysis, OA was calculated by computing the number of observations with complete agreement and dividing this number with the total number of anatomical sites assessed. The three-reader AC was calculated by multiplication of the marginal fractions [27]. Reliability measures were computed using unweighted kappa statistics owing to the categorical (as opposed to ordinal) nature of the data. Given the condition of total independence among the readers, Κ is defined as

$$ \mathrm{K}=\frac{OA- AC}{1- AC} $$

where OA is observed agreement and AC agreement by chance [29]. Reliability measures were computed for the readers in pairs (A1B1, A1C1, B1C1, A1A2) and over-all (A1B1C1). Acknowledging the influence of prevalence on the Κ estimates [27, 28], these were only computed whenever the readers in question agreed on prevalences ≥10%. For each disc level, the left and right hand side assessments of neural foraminal stenosis, uncovertebral and zygapophyseal osteoarthritis were pooled before computing reliability estimates. The interpretation of Κ values followed the suggestions by Landis & Koch [29]:

**Table:** 

*Κ value*	*Strength of agreement*
< 0.0:	Poor
0.0–0.2	Slight
0.21–0.4	Fair
0.41–0.6	Moderate
0.61–0.8	Substantial
0.81–1.0	Almost perfect

Κ values were reported using 95% confidence intervals and additional information on OA and AC were supplied for all findings. Analyses were performed using the STATA (version 15.0; Stata Corporation, College Station, Texas, USA) software package.

### Ethics

All subjects provided written informed consent. The study was approved by the Regional Data Protection Agency (J.no. 1–16–02-86-16). Approval by the regional ethical committee was not needed due to the study’s methodological nature. The letter of exemption from The Central Denmark Region Committees on Health Research Ethics is available from the author on request (case no. 86 / 2017).

## Results

The majority of the subjects were female (*n* = 31; 62%) with a mean age of 43.7 years (SD = 9.2). The prevalence of positive findings for all readers can be seen in Additional file 2. For vertebral endplate signal changes, prevalence estimates were below 10% and thus too low for Κ statistics. For the remaining degenerative findings, prevalence estimates allowed for kappa statistics including one to six anatomical sites (e.g. 2 disc levels ~ 100 observations included in Κ analysis for spinal canal stenosis). Further scrutiny of the prevalence table revealed a slight tendency for Reader C to assign the label “reduced disc height” more frequently. Otherwise no systematic differences among the readers were identified.

As shown in Table 2, the overall inter-rater reliability (A1B1C1) ranged from moderate to almost perfect for the majority of the findings (substantial to almost perfect for kyphosis and neural foraminal stenosis; moderate to almost perfect for spinal canal stenosis; and moderate to substantial for disc height, disc contour, uncovertebral and zygapophyseal osteoarthritis). Exploratory analyses were made to assess the inter-rater reliability of neural foraminal stenosis when including only MRIs with oblique images (Additional file 3). This did not change the reliability estimates but broadened the confidence intervals slightly.

**Table 2:** Inter-rater reliability estimates

MRI finding	n	Reader pair	Observed agreement (%)	Agreement by chance (%)	Kappa (95% CI)
Kyphosis^a^	50	A1B1	92.0	56.4	0.82 (0.75; 0.89)
	49	A1C1	89.8	53.6	0.78 (0.71; 0.85)
	49	B1C1	89.8	52.8	0.78 (0.71; 0.86)
	49	A1B1C1	85.7	31.2	0.79 (0.73; 0.85)
Disc height^b^	150	A1B1	92.0	52.8	0.83 (0.74; 0.92)
	200	A1C1	80.0	52.8	0.58 (0.46; 0.69)
	150	B1C1	77.3	50.0	0.55 (0.42; 0.68)
	150	A1B1C1	74.7	26.4	0.65 (0.57; 0.74)
Disc contour^b^	177	A1B1	76.8	43.4	0.59 (0.49; 0.70)
	177	A1C1	79.7	43.3	0.64 (0.53; 0.74)
	200	B1C1	80.0	47.6	0.62 (0.52; 0.72)
	177	A1B1C1	68.4	21.7	0.61 (0.54; 0.69)
Spinal canal stenosis^b^	100	A1B1	97.0	76.0	0.88 (0.68; 1.00)
	100	A1C1	91.0	73.5	0.66 (0.47; 0.83)
	100	B1C1	92.0	74.3	0.69 (0.48; 0.86)
	100	A1B1C1	90.0	63.0	0.74 (0.57; 0.86)
Vertebral endplate signal change	Too low prevalences (i.e. ≤ 10%)				
Uncovertebral osteoarthritis^c^	222	A1B1	90.1	68.0	0.69 (0.57; 0.81)
	237	A1C1	89.0	68.6	0.65 (0.53; 0.77)
	230	B1C1	87.4	70.9	0.57 (0.43; 0.71)
	222	A1B1C1	83.3	53.0	0.65 (0.51; 0.76)
Zygapophyseal osteoarthritis^c^	270	A1B1	94.8	74.2	0.80 (0.70; 0.90)
	144	A1C1	87.5	74.9	0.50 (0.31; 0.70)
	184	B1C1	85.9	78.9	0.33 (0.13; 0.53)
	135	A1B1C1	83.0	61.0	0.56 (0.43; 0.70)
Neural foraminal stenosis^c^	268	A1B1	90.7	64.1	0.74 (0.65; 0.84)
	287	A1C1	90.2	64.2	0.73 (0.63; 0.82)
	275	B1C1	87.6	65.8	0.64 (0.53; 0.75)
	268	A1B1C1	84.0	46.0	0.73 (0.63; 0.82)

^a^n refers to the number of MRIs assessed

^b^n refers to the number of disc levels assessed

^c^n refers to the number of anatomical sites assessed (by pooling right and left hand side)

The intra-rater reliability estimates (Table 3) were slightly better than those for inter-rater reliability. Almost perfect reliability was found for kyphosis and substantial to almost perfect reliability for disc contour, uncovertebral osteoarthritis and neural foraminal stenosis. For spinal canal stenosis and zygapophyseal osteoarthritis, moderate to almost perfect intra-rater reliability was found while moderate to substantial reliability was found for disc height.

**Table 3:** Intra-rater reliability estimates

MRI finding	n	Reader pair	Observed agreement (%)	Agreement by chance (%)	Kappa (95% CI)
Kyphosis^a^	50	A1A2	96.0	59.6	0.90 (0.85; 0.96)
Disc height^b^	200	A1A2	84.0	51.5	0.67 (0.57; 0.77)
Disc contour^b^	174	A1A2	88.5	43.9	0.80 (0.71; 0.87)
Spinal canal stenosis^b^	50	A1A2	94.0	76.6	0.73 (0.51; 0.90)
Vertebral endplate signal change	Too low prevalences (i.e. ≤ 10%)				
Uncovertebral osteoarthritis^c^	281	A1A2	90.4	67.0	0.71 (0.61; 0.81)
Zygapophyseal osteoarthritis^c^	240	A1A2	90.8	68.8	0.71 (0.59; 0.82)
Neural foraminal stenosis^c^	287	A1A2	90.6	62.6	0.75 (0.66; 0.84)

^a^n refers to the number of MRIs assessed

^b^n refers to the number of disc levels assessed

^c^n refers to the number of anatomical sites assessed (by pooling right and left hand side)

## Discussion

To our knowledge, this is the first reliability study covering eight common cervical MRI findings. The overall inter-rater reliability was substantial for all variables except zygapophyseal osteoarthritis where moderate reliability was found. Intra-rater reliability was substantial for the majority of variables and almost perfect for kyphosis. These reliability estimates reflect that the observed agreement notably exceeds the agreement that can be expected by chance.

For disc degeneration, other studies [9, 12] reported higher reliability estimates than the disc height estimates in the current study. Although the use of intraclass correlation coefficient in the study by Jacobs et al. [12] does not allow for direct comparison, possible explanations for the reliability differences are the use of a ubiquitously accessible reference image of a normal disc [12] and the notable experience among readers with the same educational background [9].

For disc contour, the reliability estimates were similar to those of other studies despite the fact that we used a three-category classification compared to the previously reported dichotomous classifications [8, 30, 31] and comparison of more experienced readers [30, 31].

For spinal canal stenosis, the current study’s unweighted reliability estimates exceeded those previously reported by use of weighted kappa statistics [13, 32], although the use of weights are expected to yield higher estimates. A higher number of readers (six [13] and nine [32]) could explain this difference, but even when compared to the three most experienced readers in these studies, better reliability estimates were still achieved in the current study. The most probable reason appears to be the limited introduction of their classification [13, 32]. When using both written and visual descriptions, our moderate to almost perfect reliability among readers with considerable experience differences suggest good applicability of this classification of spinal canal stenosis.

For zygapophyseal osteoarthritis, both the intra- and inter-rater reliability estimates were better than previously reported [11], which is most likely explained by the use of a dichotomous variable in the current study compared to a classification with four severity categories [11].

For neural foraminal stenosis, this study still achieved higher reliability estimates compared to studies with more experienced readers [30, 31]. The inferior reliability estimates may be explained by unclear definitions [30] and by low prevalence estimates together with images obtained using a 0.5 T field strength [31]. Compared to the study from which we modified the classification of neural foraminal stenosis [10], the current study was unable to reach the same almost perfect reliability estimates (Κ > 0.9). Nevertheless, we consider the substantial to almost perfect reliability to be satisfactory, bearing in mind differences in reader experience and the heterogeneous image material (i.e. images with different field strengths and available sequences). The modified classification (dichotomous versus the original four categories) proved reliable and the association with clinical findings has previously been reported [33].

### Methodological considerations

A limitation of the study is that it was not preceded by a power calculation. However; the confidence intervals for the Κ estimates only comprised more than two levels (e.g. from moderate to almost perfect for spinal canal stenosis) in a minority of cases. A larger sample would have narrowed the confidence intervals but would probably not have caused substantial changes in the reliability estimates.

Another limitation is the involvement of only reader A in the intra-rater analysis. Two considerations explain this: 1) previous reliability studies found higher [7–9, 12, 14, 21] or similar/higher [10, 11, 13] intra-rater reliability than inter-rater reliability and 2) involvement of reader A was necessary since a future prognostic study will involve MRI assessments performed by reader A. As for the inter-rater reliability, the study included three readers, only one of these being a radiologist. However, the results suggest that our method is applicable among other health care professionals (i.e. rheumatologists and chiropractors) in a controlled research setting. Involvement of other relevant healthcare professionals, e.g. spine surgeons, would have been desirable but was unfortunately not possible.

Owing to the properties of Κ, the measure does not disentangle systematic and random misclassification [28]. Therefore, we provided the prevalence tables from which we find no suspicion of systematic misclassification.

The prevalence table discloses a notable difference in the number of disc levels assessed for disc contour on levels C2/C3, C3/C4 and C7/T1: Reader A assessed fewer levels than Readers B and C owing to the lack of axial images of the selfsame disc levels. This discrepancy suggests a difference among the readers, and whether this partly explains why higher reliability estimates were not achieved for disc contour cannot be refuted.

Another potential limitation is that all MRIs were derived only from individuals with neck pain. But since cervical spine MRI is seldom performed in patients without neck pain and since the future use of the evaluation manual applies to patients with neck pain, we consider the current sample appropriate for its purpose.

Finally, a potential limitation of the study is the heterogeneous image material (MRIs were performed at five different hospitals. Different field strengths and sequences were available). Yet, as it resembles everyday clinical practice, this was an intended challenge and an attempt was made to manage this heterogeneity by using a standardized evaluation manual. The differences between OA and AC (Tables 2 and 3) reflect that both inter- and intra-rater agreement notably exceed the agreement that can be expected by chance. Furthermore, the high levels of observed agreement reflect only a minor degree of misclassification. Based on these observations of OA, our interpretation is that the evaluation manual and the standardized procedures explain the high levels of agreement rather than pure chance when assessing heterogeneous images.

Ultimately, the heterogeneous image material and the use of three different health care professionals both add to the generalizability and thus constitute strengths of the study. The blinding of the readers, the use of simple and easily comprehensible classifications along with regular encouragement to follow the evaluation manual, are other important strengths of the study.

In contrast to the controlled settings of the current study, a study comparing narrative MRI reports demonstrated considerable variability [34]. In this study [34], a patient with low back pain and right L5 radicular symptoms had lumbar spine MRI performed at 10 different MRI centers within 3 weeks. Comparison of the 10 narrative reports revealed considerable variability; none of the 49 described findings occurred in all 10 reports and only one finding occurred in nine reports. Even if this amount of variability is unusually large [34], it supports our clinical experience that variability also prevails in the interpretation of cervical spine MRIs. A possible way to overcome this is by using classifications sufficiently comprehensible to be applied 1) by different health care professionals and 2) when assessing heterogeneous images from different MRI scanners. Such classifications were presented in the current study. Confirmatory studies will be needed. If those studies were to involve experienced radiologists, provide proper training for lesser experienced MRI readers, and use an evaluation manual, better reliability might be achieved in clinical settings. So far, the results suggest that the evaluation of MRI findings can be used in controlled research settings studying individuals with neck pain. Suggestions for future research include comparison of reliability with and without the use of an evaluation manual. Also, including more than one of each health care professional could allow for comparison of experience levels both among and within different types of health care professionals.

## Conclusions

In conclusion, the current study found substantial reliability for the majority of included MRI findings. This suggests that the present classifications are sufficiently comprehensible to be applied by different health care professionals when assessing images from different MRI scanners. In our view, the proposed classifications are sufficiently reliable to be used for both quality assurance and further research purposes.

## Additional files

Additional file 1: The evaluation manual used for assessment of the MRIs. (DOCX 2347 kb)

Additional file 2: A prevalence table reporting the frequency of positive findings for all the readers. (DOCX 30 kb)

Additional file 3: A table of sensitivity analyses. For neural foraminal stenosis, kappa estimates are presented comparing the assessments of all images vs. only images with available oblique slices. (DOCX 16 kb)

## Abbreviations

AC: Agreement by chance
CSF: Cerebrospinal fluid
GP: General practitioner
MRI: Magnetic resonance imaging
OA: Observed agreement
RCT: Randomized controlled trial
SD: Standard deviation
Κ: Kappa
