# Supplementary material for: Degenerative findings on MRI of the cervical spine: an inter- and intra-rater reliability study
Source: Chiropr Man Therap. 2018 Oct 16;26:43. doi: 10.1186/s12998-018-0210-2 (PMC6190655; doi:10.1186/s12998-018-0210-2)
Supplement: Supplementary file 1 — The evaluation manual used for assessment of the MRIs. (DOCX 2347 kb) [file 12998_2018_210_MOESM1_ESM.docx]

Evaluation manual for the inter- and intra-rater reliability study on MRI of the cervical spine

This evaluation manual covers all the definitions and classifications that are used for the above mentioned reliability study.

In this evaluation manual, the names of the variables are so-called 'generic' names, whereas variables in Epidata have appropriate prefixes to determine the cervical level in question. Likewise, for the neural foramina, the uncovertebral and zygapophyseal joints, suffixes in Epidata are used to denote whether it is on the left or the right hand side.

Six disc levels are examined from C2/C3 to C7/T1.

____________________________________________________________________

**Kyphosis**

0 : normal or reduced lordosis

1 : kyphosis

9 : missing

Cervical alignment is assessed on mid-sagittal T2-weighted images. A line from the posteroinferior aspect of vertebra C2 to the posteroinferior aspect of vertebra C7 is drawn. If any part of C3-C6 lie behind/posterior to this line, the alignment is classified as kyphosis (1).

____________________________________________________________________

**Disc height**

0 : normal disc height

1 : reduced disc height

9 : missing

Disc height is assessed on T2-weighted sagittal images.

This classification is inspired by Jacobs et al. (2) but modified since the relatively small anatomical structures in the cervical spine complicate assessments of nucleus signal.

Reduced height is assessed by comparison with neighbouring discs that look morphologically normal (3). In case of extensive disc degeneration thus complicating between-disc comparison, the assessment is based on the experience of the reader.


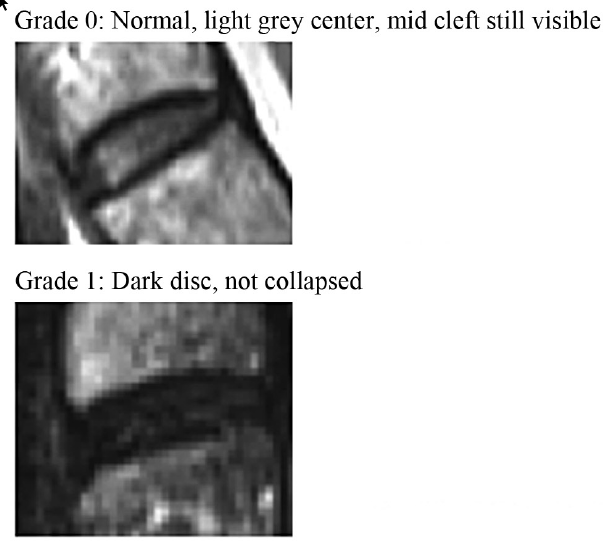


Both of these have normal disc height = grade 0. From Jacobs et al. (2).


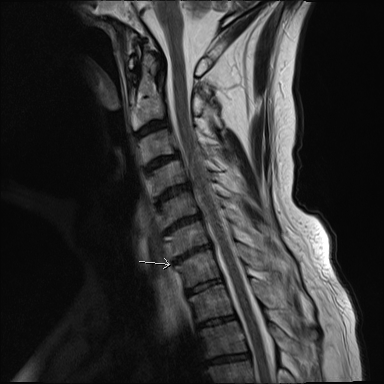


Reduced height = grade 1


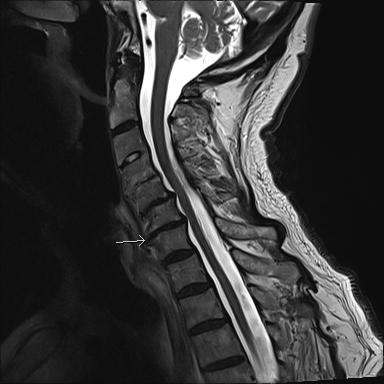


Reduced height = grade 1

__________________________________________________________

**Disc contour**

0 : normal

1 : bulge or protrusion

2 : extrusion

9 : missing

Disc contour is assessed by use of both sagittal and axial images. We use a modified version of the classification by Fardon et al. 2014 (4).

- A *normal* disc is defined as *morphologically* normal. I.e., there is no sign of illness, trauma or ageing. The disc is located within the "disc space", the limits of which are determined by the vertebral endplates craniocaudally. The peripheral limits of "disc space" are constituted by the apophyses of the vertebrae (excluding possible osteophytes) (4).
- *Disc bulge describes a general (in some cases asymmetric) bulging of disc material. The material has moved beyond* "disc space" and comprises > 25% of the disc circumference. Usually, a bulge does not exceed 3 mm beyond "disc space" (4).
- *Protrusion:* A focal displacement of disc material of which the base involves < 25% of the circumference. The maximum measure of the displaced disc material is smaller than the measure of the base of the displaced material at the disc space of origin, measured in the same plane (4).
- *Extrusion:* A focal displacement of disc material where the base involves

< 25% of the disc circumference. The maximal measure of the displaced disc material is greater than the measure of the base of the displaced material at the disc space of origin, measured in the same plane. Extrusion also comprises what was formerly denoted as sequestration where the displaced material has lost continuity with the disc space of origin (4).


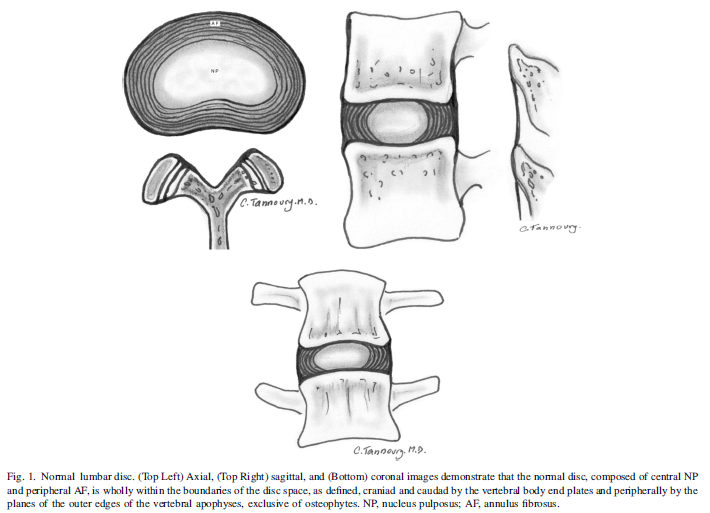


**Normal discs (4)**

**Normal discs on axial and sagittal images**


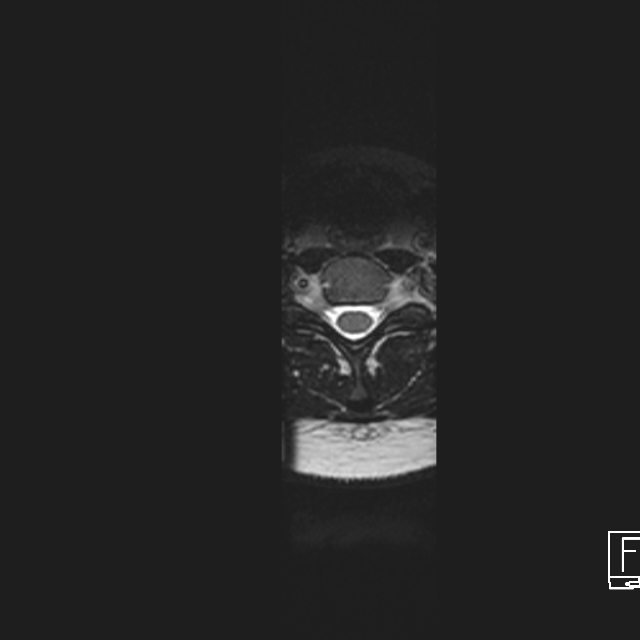

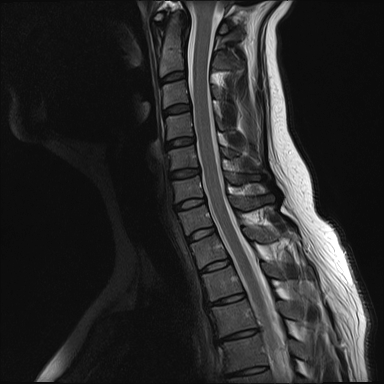


______________________________

**Bulge (4)**


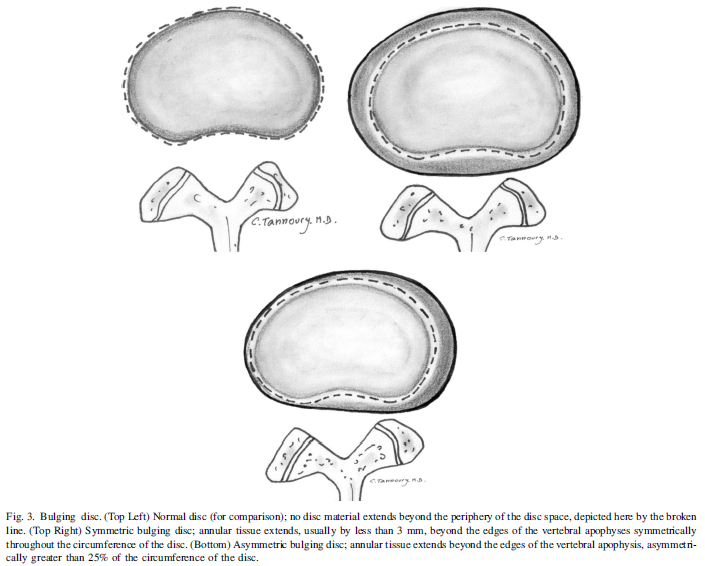


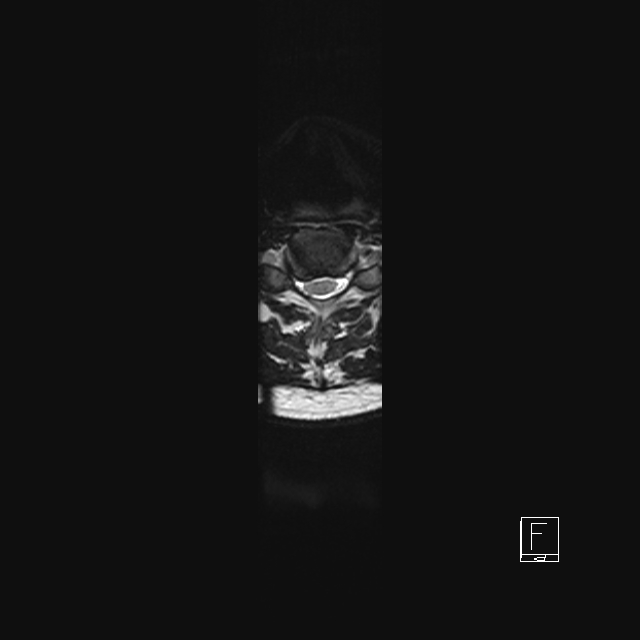


**Bulge**

___________________________________


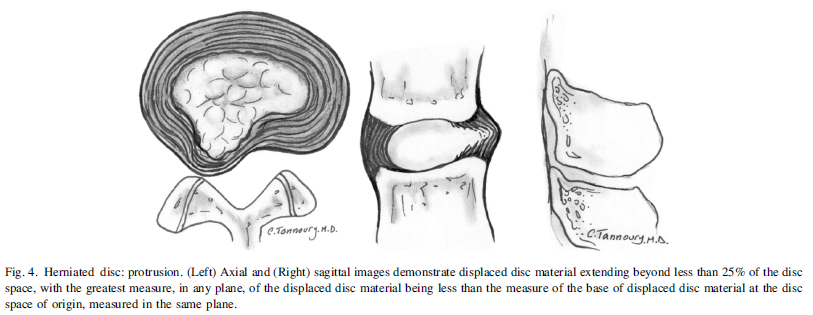


# Protrusion (4)


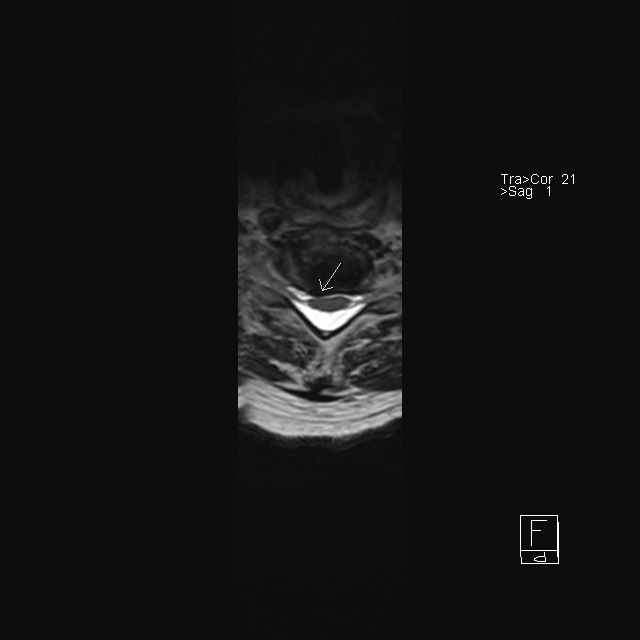


# Protrusion, axial


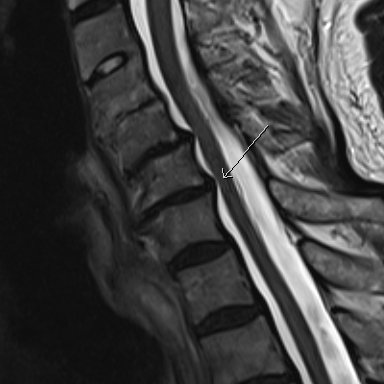


# Protrusion, sagittal

_______________________________________________


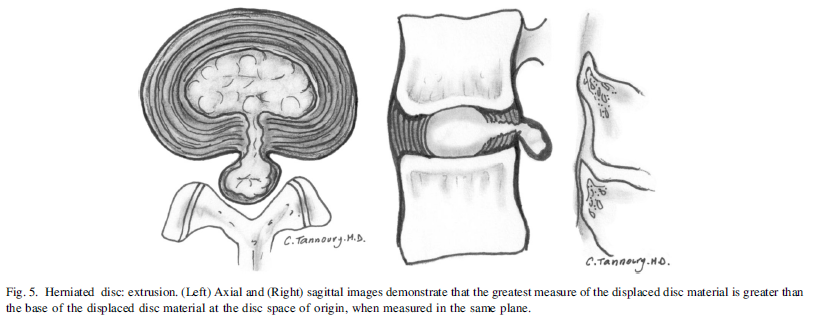


# Extrusion (4)


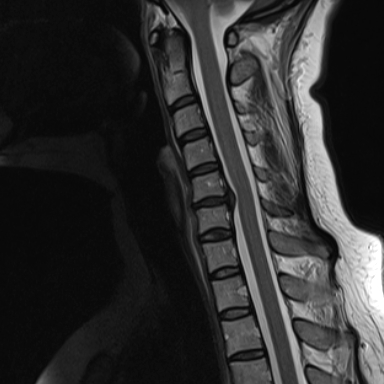


# Extrusion, sagittal


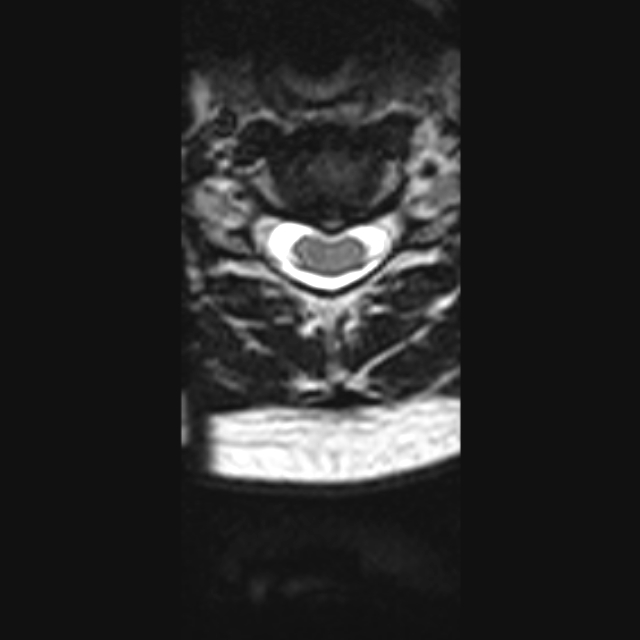


# Extrusion, axial

_______________________________________________________________

**Spinal canal stenosis**

0 : no stenosis

1 : >50% obliteration of CSF, no cord deformity

2 : >50% obliteration of CSF with cord deformity but no signal change

3 : >50% obliteration of CSF with cord deformity and signal change

9 : missing

The spinal canal is assessed on three T2-weighted images; the mid-sagittal and the two neighbouring slices (one on each side of the mid-sagittal image). The classification by Kang et al. is used (5).

# Grade 0-3 (5):


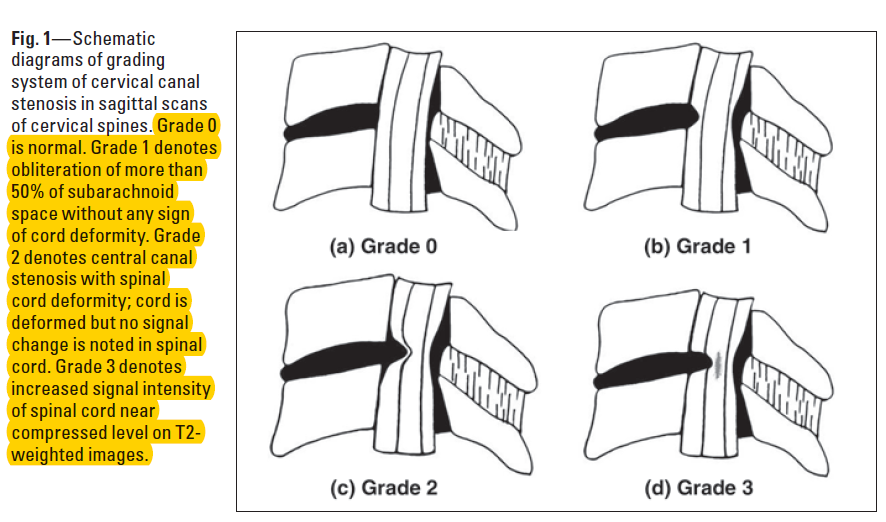


________________________________________________________________

**Vertebral endplate signal change (VESC)**

| 0 : normal |
| --- |
| 1 : VESC type 1 |
| 2 : VESC type 2 |
| 3 : VESC type 3 |
| 4 : mixed VESC type 1 and 2 |

9 : missing

Primarily assessed on sagittal images. VESC constitute changes in the endplates of two neighbouring vertebral endplates surrounding a degenerated disc (4). If only visible on one slice, the finding is excluded (6). Likewise, changes only related to osteophytes or Schmorl nodes will be excluded.

*Type 1* is hyperintense on T2-weighted and hypo-/isointense on T1-weighted images (7).

*Type 2* is hyperintense on T1-weighted images and hyper-/isointense on T2-weighted images (7).

*Type 3* er hypointense on T1 og T2 (bone sclerosis where hydrogen protons are so tightly bound and thus difficult to bring in motion 🡪 no MR signal on T1 and T2) (4).


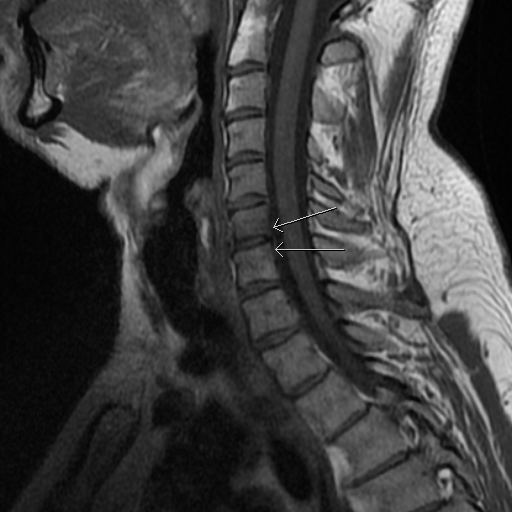


# VESC type 1. T1-weighted


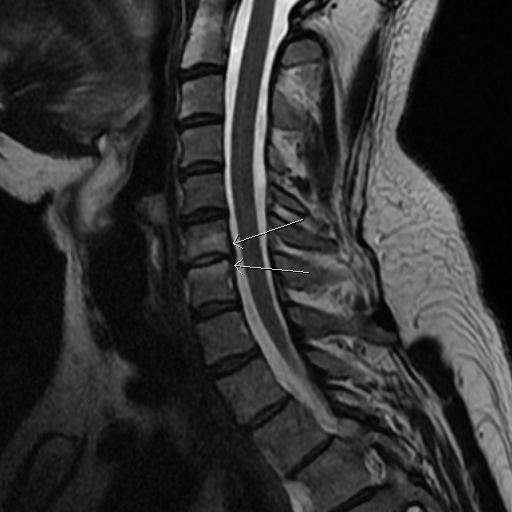


# VESC type 1. T2-weighted


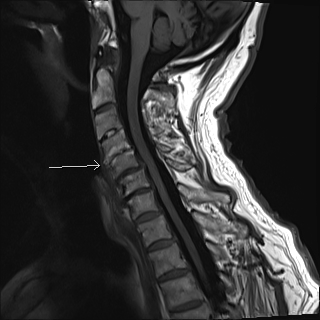


# VESC type 2. T1-weighted


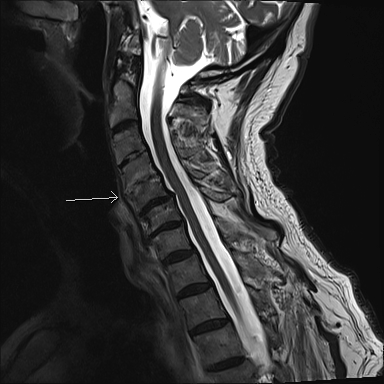


# VESC type 2. T2-weighted

**Drawing of type 1, 2 og 3** **(4)**


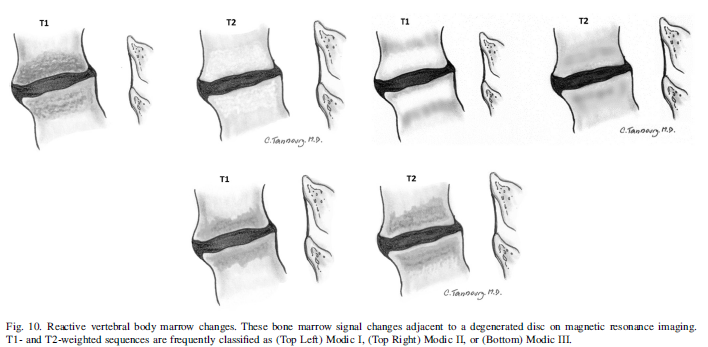


____________________________________________________________________

**Neural foraminal stenosis**

0 : normal or < 50% obliteration of perineural fat

1 : >= 50% obliteration of perineural fat with/without nerve root compromise.

9 : missing

The extent of perineural fat obliteration is determined on T2 weighted oblique images. The assessment is made on the slice with most severe stenosis. Meanwhile, on the axial images, the reader ensures that he/she is actually looking at the neural foramen. The boundaries of the neural foramen are: to the anterior the disc and posterior aspect of the vertebral bodies; to the posterior the zygapophyseal joint; and cranially and caudally the pediculi (8). On axial images, the medial and lateral boundaries are the medial and lateral aspect of the pediculi, respectively (9).

If no oblique images are available, the assessment is based on the axial images where stenosis is most severe, i.e. where the distance between the processus articularis superior (posterolaterally) and corpus vertebra/discus (anteromedially) is identified (10).


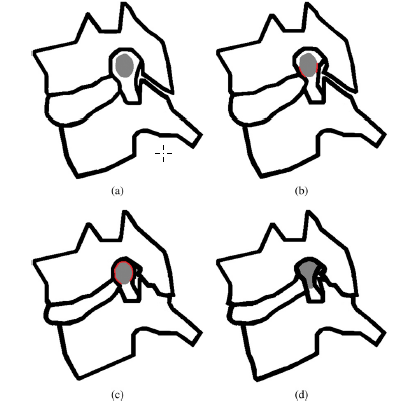


# Grade 1(15):

**1 = figure c+d**

# Grade 0 (15):

**0 = figure a +b**


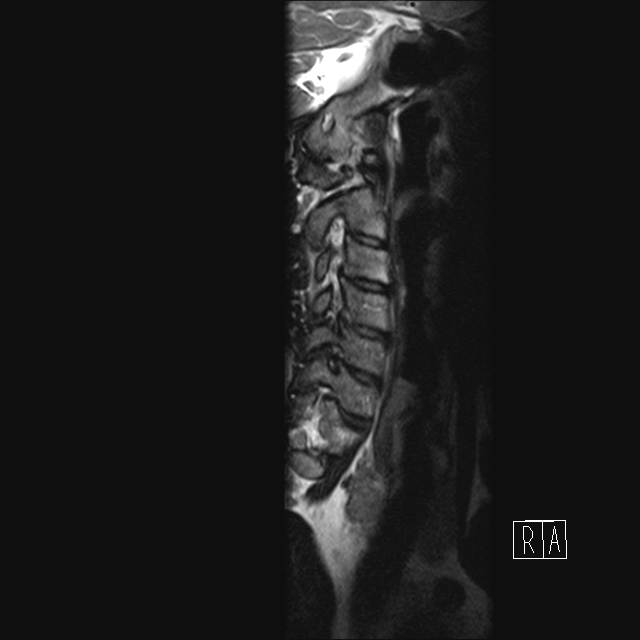


# Grade 1

# In the following, foraminal stenosis on axial images is depicted. While assessing the axial images, the sagittal images are used to demonstrate the location in relation to the processes articulares. The severity of stenosis is determined by comparing the relevant number of subsequent axial slices in the neural foramen in question.


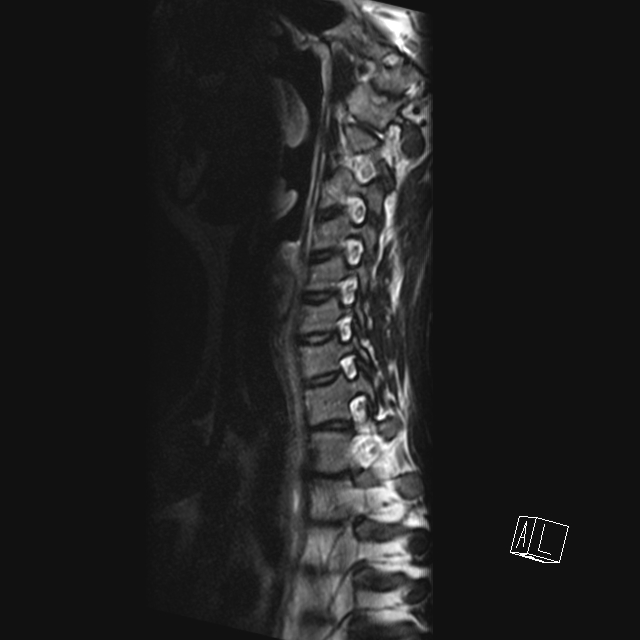


# Grade 0

____________________________________________________________________

**Uncovertebral osteoarthritis**

0 : normal

1 : definite osteoarthritis (primarily assessed by the presence of osteophytes which take up place in the neural foramen).

9 : missing

**Only** if >= 50% foraminal stenosis is settled on, the reader can consider the classification definite osteoarthritis. (i.e. if foraminal stenosis is <50%, any possible irregularities of the uncinate process are not articulate enough to be classified as osteoarthritis). The classification was chosen because degenerative changes of the uncovertebral joint primarily comprise the growth of osteophytes (11).

Assessment is only done on oblique images which allow for the best assessments (11,12). If these are not available, the value '9' = missing is used due to the risk of partial volume effect on axial images.


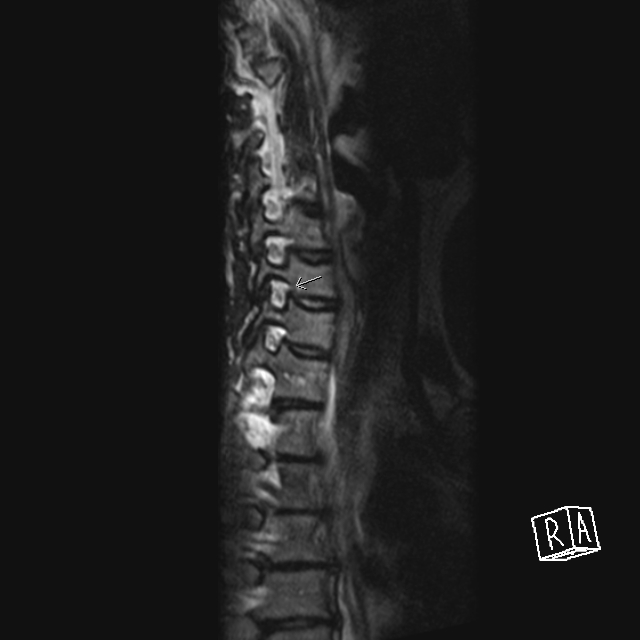


# Grade 0, normal uncovertebral joint


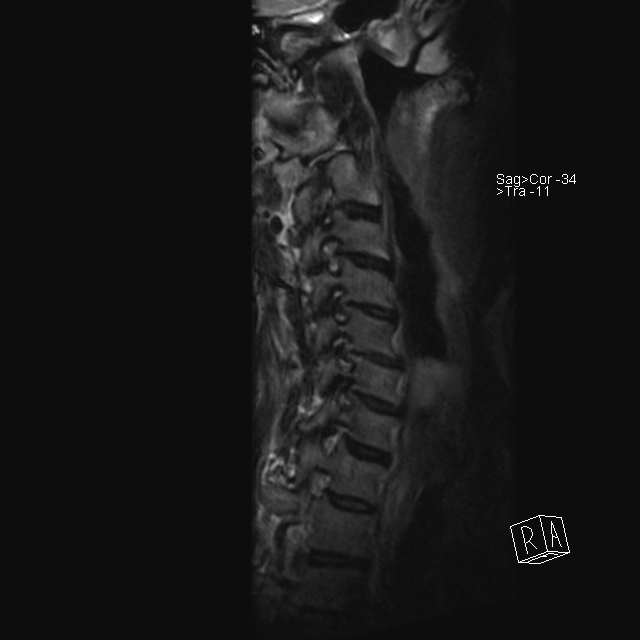


# Grade 1, definite uncovertebral osteoarthritis

____________________________________________________________________

**Zygapophyseal osteoarthritis**

0 : normal (no definite joint space narrowing, osteophytes or hypertrophy of the processus articularis)

1 : definite osteoarthritis (definite joint space narrowing, osteophytes or hypertrophy of the processus articularis) (13,14)

9 : missing

The assessment is done on oblique images (12). If these are not availabe, axial and sagittal images are used.

**Only** if >= 50% foraminal stenosis is settled on, the reader can consider the classification definite osteoarthritis. (i.e. if foraminal stenosis is <50%, any possible irregularities of the zygapophyseal joint are not articulate enough to be classified as osteoarthritis.


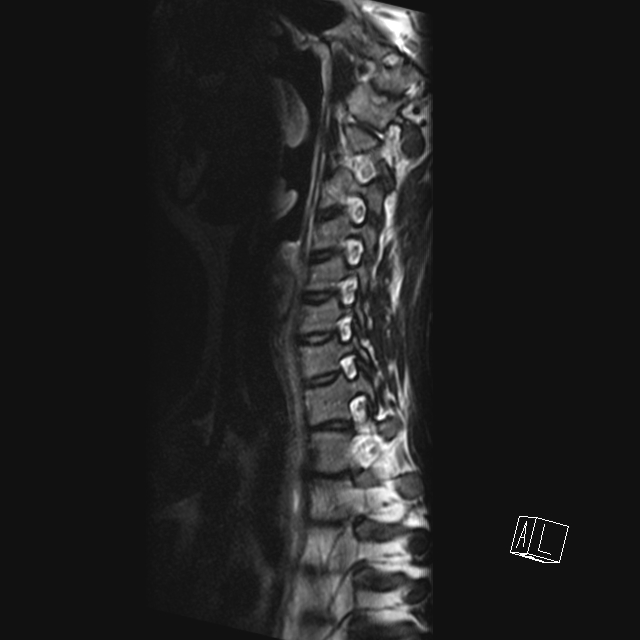


# Grade 0, normal zygapophyseal joint


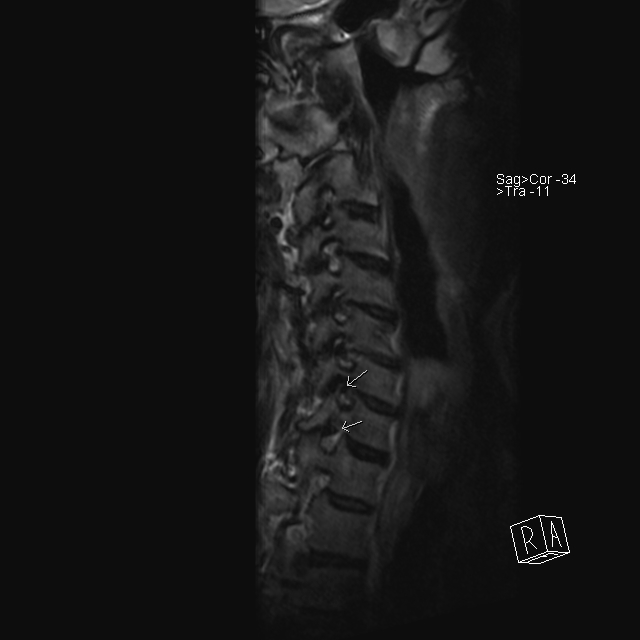


# Grade 1, definite zygapopyseal osteoarthritis

____________________________________________________________________

**Comments**

If any other relevant pathological findings are identified. This may be spondylolistesis, fracture, cord pathology etc. ____________________________________________________________________

(1) Nouri A, Martin AR, Mikulis D, Fehlings MG. Magnetic resonance imaging assessment of degenerative cervical myelopathy: a review of structural changes and measurement techniques. Neurosurg Focus 2016 Jun;40(6):E5.

(2) Jacobs LJ, Chen AF, Kang JD, Lee JY. Reliable Magnetic Resonance Imaging Based Grading System for Cervical Intervertebral Disc Degeneration. Asian Spine J 2016 Feb;10(1):70-74.

(3) Fu MC, Webb ML, Buerba RA, Neway WE, Brown JE, Trivedi M, et al. Comparison of agreement of cervical spine degenerative pathology findings in magnetic resonance imaging studies. Spine J 2016 Jan 1;16(1):42-48.

(4) Fardon DF, Williams AL, Dohring EJ, Murtagh FR, Gabriel Rothman SL, Sze GK. Lumbar disc nomenclature: version 2.0: Recommendations of the combined task forces of the North American Spine Society, the American Society of Spine Radiology and the American Society of Neuroradiology. Spine J 2014 Nov 1;14(11):2525-2545.

(5) Kang Y, Lee JW, Koh YH, Hur S, Kim SJ, Chai JW, et al. New MRI grading system for the cervical canal stenosis. AJR Am J Roentgenol 2011 Jul;197(1):W134-40.

(6) Maatta JH, Karppinen J, Paananen M, Bow C, Luk KD, Cheung KM, et al. Refined Phenotyping of Modic Changes: Imaging Biomarkers of Prolonged Severe Low Back Pain and Disability. Medicine (Baltimore) 2016 May;95(22):e3495.

(7) Modic MT, Steinberg PM, Ross JS, Masaryk TJ, Carter JR. Degenerative disk disease: assessment of changes in vertebral body marrow with MR imaging. Radiology 1988 Jan;166(1 Pt 1):193-199.

(8) Bojsen-Moeller F. Chapter 8: Hvirvelsoejlen (The Spine). Bevaegeapparatets Anatomi. 12th ed. Copenhagen: Munksgaard Danmark; 2001. p. 89.

(9) Wiltse LL, Berger PE, McCulloch JA. A system for reporting the size and location of lesions in the spine. Spine (Phila Pa 1976) 1997 Jul 1;22(13):1534-1537.

(10) Kim S, Lee JW, Chai JW, Yoo HJ, Kang Y, Seo J, et al. A New MRI Grading System for Cervical Foraminal Stenosis Based on Axial T2-Weighted Images. Korean J Radiol 2015 Nov-Dec;16(6):1294-1302.

(11) Yochum TR, Rowe LJ. Chapter 10: Arthritic Disorders. Essentials of Skeletal Radiology; 1996. p. 795-807.

(12) Shim JH, Park CK, Lee JH, Choi JW, Lee DC, Kim DH, et al. A comparison of angled sagittal MRI and conventional MRI in the diagnosis of herniated disc and stenosis in the cervical foramen. Eur Spine J 2009 Aug;18(8):1109-1116.

(13) Kalichman L, Suri P, Guermazi A, Li L, Hunter DJ. Facet orientation and tropism: associations with facet joint osteoarthritis and degeneratives. Spine (Phila Pa 1976) 2009 Jul 15;34(16):E579-85.

(14) Xu C, Ding ZH, Xu YK. Comparison of computed tomography and magnetic resonance imaging in the evaluation of facet tropism and facet arthrosis in degenerative cervical spondylolisthesis. Genet Mol Res 2014 May 30;13(2):4102-4109.

(15) Park HJ, Kim SS, Lee SY, Park NH, Chung EC, Rho MH, et al. A practical MRI grading system for cervical foraminal stenosis based on oblique sagittal images. Br J Radiol 2013 May;86(1025):20120515.
