# Supplementary material for: Degenerative findings on MRI of the cervical spine: an inter- and intra-rater reliability study
Source: Chiropr Man Therap. 2018 Oct 16;26:43. doi: 10.1186/s12998-018-0210-2 (PMC6190655; doi:10.1186/s12998-018-0210-2)
Supplement: Supplementary file 2 — A prevalence table reporting the frequency of positive findings for all the readers. (DOCX 30 kb) [file 12998_2018_210_MOESM2_ESM.docx]

| Prevalence of positive findings | | | | | | | | |
| --- | --- | --- | --- | --- | --- | --- | --- | --- |
|  | Reader A, 1st assessment | | Reader B | | Reader C | | Reader A, 2nd assessment | |
| MRI finding | Number of MRIs assessed | Positive findings  n (%) | Number of MRIs assessed | Positive findings  n (%) | Number of MRIs assessed | Positive findings  n (%) | Number of MRIs assessed | Positive findings  n (%) |
| Kyphosis | 50 | 15 (30.0) | 50 | 17 (34.0) | 50 | 20 (40.0) | 50 | 13 (26.0) |
|  | | | | | | | | |
| Disc height C2/C3 | 50 | 3 (6.0) | 50 | 0 (0.0) | 50 | 1 (2.0) | 50 | 3 (6.0) |
| Disc height C3/C4 | 50 | 8 (16.0) | 50 | 4 (8.0) | 50 | 8 (16.0) | 50 | 10 (20.0) |
| Disc height C4/C5 | 50 | 12 (24.0) | 50 | 15 (30.0) | 50 | 21 (42.0) | 50 | 17 (34.0) |
| Disc height C5/C6 | 50 | 26 (52.0) | 50 | 24 (48.0) | 50 | 32 (64.0) | 50 | 36 (72.0) |
| Disc height C6/C7 | 50 | 21 (42.0) | 50 | 16 (32.0) | 50 | 22 (44.0) | 50 | 28 (56.0) |
| Disc height C7/T1 | 50 | 1 (2.0) | 50 | 1 (2.0) | 50 | 2 (4.0) | 50 | 3 (6.0) |
|  |  | | | | | | | |
| Disc contour C2/C3 | 30 | 1 (3.3) | 50 | 1 2.00 | 50 | 1 (2.0) | 31 | 1 (3.2) |
| Disc contour C3/C4 | 38 | 11 (28.9) | 50 | 9 (18.0) | 50 | 8 (16.0) | 35 | 9 (25.7) |
| Disc contour C4/C5 | 45 | 15 (33.3) | 50 | 13 (26.0) | 50 | 15 (30.0) | 45 | 16 (35.6) |
| Disc contour C5/C6 | 48 | 31 (64.6) | 50 | 26 (52.0) | 50 | 32 (64.0) | 48 | 35 (72.9) |
| Disc contour C6/C7 | 46 | 26 (56.5) | 50 | 19 (38.0) | 50 | 24 (48.0) | 46 | 26 (56.5) |
| Disc contour C7/T1 | 38 | 2 (5.2) | 50 | 3 (6.0) | 50 | 1 (2.0) | 34 | 2 (5.9) |
|  | | | | | | | | |
| Spinal canal stenosis C2/C3 | 50 | 0 (0.0) | 50 | 0 (0.0) | 49 | 1 (2.0) | 50 | 0 (0.0) |
| Spinal canal stenosis C3/C4 | 50 | 1 (2.0) | 50 | 1 (2.0) | 49 | 1 (2.0) | 50 | 1 (2.0) |
| Spinal canal stenosis C4/C5 | 50 | 1 (2.0) | 50 | 1 (2.0) | 50 | 5 (10.0) | 50 | 1 (2.0) |
| Spinal canal stenosis C5/C6 | 50 | 7 (14.0) | 50 | 6 (12.0) | 50 | 8 (16.0) | 50 | 6 (12.0) |
| Spinal canal stenosis C6/C7 | 50 | 7 (14.0) | 50 | 7 (14.0) | 50 | 8 (16.0) | 50 | 5 (10.0) |
| Spinal canal stenosis C7/T1 | 50 | 0 (0.0) | 50 | 0 (0.0) | 50 | 0 (0.0) | 50 | 0 (0.0) |
|  | | | | | | | | |
| VESC C2/C3 | 50 | 0 (0.0) | 50 | 1 (2.0) | 50 | 0 (0.0) | 50 | 0 (0.0) |
| VESC C3/C4 | 50 | 1 (2.0) | 50 | 1 (2.0) | 50 | 1 (2.0) | 50 | 1 (2.0) |
| VESC C4/C5 | 50 | 0 (0.0) | 50 | 1 (2.0) | 50 | 1 (2.0) | 50 | 0 (0.0) |
| VESC C5/C6 | 50 | 2 (4.0) | 50 | 2 (4.0) | 50 | 3 (6.0) | 50 | 3 (6.0) |
| VESC C6/C7 | 50 | 3 (6.0) | 50 | 4 (8.0) | 50 | 5 (10.0) | 50 | 5 (10.0) |
| VESC C7/T1 | 50 | 0 (0.0) | 50 | 0 (0.0) | 50 | 0 (0.0) | 50 | 0 (0.0) |
| VESC: vertebral endplate signal change (Modic change) | | | | | | | | |

| Prevalence of positive findings continued | | | | | | | | | |
| --- | --- | --- | --- | --- | --- | --- | --- | --- | --- |
|  | Reader A, 1st assessment | | Reader B | | Reader C | | Reader A, 2nd assessment | | |
| MRI finding | Number of MRIs assessed | Positive findings  n (%) | Number of MRIs assessed | Positive findings  n (%) | Number of MRIs assessed | Positive findings  n (%) | Number of MRIs assessed | | Positive findings  n (%) |
| Right uncovertebral osteoarthritis C2/C3 | 44 | 1 (2.3) | 43 | 0 (0.0) | 48 | 0 (0.0) | 43 | | 0 (0.0) |
| Right uncovertebral osteoarthritis C3/C4 | 47 | 1 (2.1) | 44 | 0 (0.0) | 48 | 3 (6.3) | 46 | | 2 (4.3) |
| Right uncovertebral osteoarthritis C4/C5 | 47 | 9 (19.1) | 46 | 5 (10.9) | 49 | 6 (12.2) | 46 | | 5 (10.9) |
| Right uncovertebral osteoarthritis C5/C6 | 47 | 12 (25.5) | 46 | 8 (17.4) | 49 | 12 (24.5) | 44 | | 10 (22.7) |
| Right uncovertebral osteoarthritis C6/C7 | 48 | 11 (22.9) | 46 | 10 (21.7) | 49 | 7 (14.3) | 46 | | 14 (30.4) |
| Right uncovertebral osteoarthritis C7/T1 | 45 | 0 (0.0) | 46 | 0 (0.0) | 49 | 0 (0.0) | 45 | | 0 (0.0) |
|  | | | | | | | | | |
| Left uncovertebral osteoarthritis C2/C3 | 44 | 0 (0.0) | 44 | 0 (0.0) | 48 | 0 (0.0) | 43 | | 0 (0.0) |
| Left uncovertebral osteoarthritis C3/C4 | 47 | 3 (6.4) | 44 | 0 (0.0) | 48 | 2 (4.2) | 46 | | 4 (8.7) |
| Left uncovertebral osteoarthritis C4/C5 | 48 | 9 (18.8) | 46 | 4 (8.7) | 49 | 3 (6.1) | 46 | | 7 (15.2) |
| Left uncovertebral osteoarthritis C5/C6 | 47 | 11 (23.4) | 46 | 9 (19.6) | 49 | 9 (18.4) | 45 | | 12 (26.7) |
| Left uncovertebral osteoarthritis C6/C7 | 48 | 8 (16.7) | 46 | 5 (10.9) | 49 | 10 (20.4) | 46 | | 10 (21.7) |
| Left uncovertebral osteoarthritis C7/T1 | 45 | 0 (0.0) | 46 | 1 (2.2) | 49 | 0 (0.0) | 43 | | 0 (0.0) |
|  | | | | | | | | | |
| Right zygapophyseal osteoarthritis C2/C3 | 46 | 1 (2.2) | 43 | 1 (2.3) | 49 | 3 (6.1) | 44 | 1 (2.2) | |
| Right zygapophyseal osteoarthritis C3/C4 | 48 | 0 (0.0) | 44 | 3 (6.8) | 49 | 1 (2.0) | 46 | 2 (4.3) | |
| Right zygapophyseal osteoarthritis C4/C5 | 48 | 6 (12.5) | 46 | 6 (13.0) | 49 | 2 (4.1) | 47 | 3 (6.4) | |
| Right zygapophyseal osteoarthritis C5/C6 | 48 | 7 (14.6) | 46 | 6 (13.0) | 49 | 5 (10.2) | 47 | 11 (23.4) | |
| Right zygapophyseal osteoarthritis C6/C7 | 48 | 10 (20.8) | 46 | 7 (15.2) | 49 | 5 (10.2) | 47 | 12 (25.5) | |
| Right zygapophyseal osteoarthritis C7/T1 | 47 | 0 (0.0) | 46 | 0 (0.0) | 49 | 0 (0.0) | 45 | 0 (0.0) | |
|  | | | | | | | | | |
| Left zygapophyseal osteoarthritis C2/C3 | 46 | 0 (0.0) | 44 | 0 (0.0) | 49 | 0 (0.0) | 43 | 0 (0.0) | |
| Left zygapophyseal osteoarthritis C3/C4 | 48 | 0 (0.0) | 44 | 0 (0.0) | 49 | 1 (2.0) | 46 | 3 (6.5) | |
| Left zygapophyseal osteoarthritis C4/C5 | 48 | 5 (10.4) | 46 | 6 (13.0) | 49 | 4 (8.2) | 48 | 8 (16.7) | |
| Left zygapophyseal osteoarthritis C5/C6 | 48 | 9 (18.8) | 46 | 7 (15.2) | 49 | 6 (12.2) | 47 | 12 (25.5) | |
| Left zygapophyseal osteoarthritis C6/C7 | 48 | 8 (16.7) | 46 | 5 (10.9) | 49 | 3 (6.1) | 47 | 10 (21.3) | |
| Left zygapophyseal osteoarthritis C7/T1 | 47 | 0 (0.0) | 46 | 0 (0.0) | 49 | 0 (0.0) | 45 | 0 (0.0) | |
|  | | | | | | | | | |
| Right neural foraminal stenosis C2/C3 | 42 | 1 (2.4) | 42 | 0 (0.0) | 46 | 3 (6.5) | 43 | 1 (2.3) | |
| Right neural foraminal stenosis C3/C4 | 46 | 1 (2.2) | 44 | 1 (2.3) | 48 | 3 (6.3) | 46 | 2 (4.3) | |
| Right neural foraminal stenosis C4/C5 | 48 | 10 (20.8) | 45 | 8 (17.8) | 49 | 8 (16.3) | 48 | 6 (12.5) | |
| Right neural foraminal stenosis C5/C6 | 48 | 13 (27.1) | 46 | 10 (21.7) | 49 | 14 (28.6) | 48 | 15 (31.3) | |
| Right neural foraminal stenosis C6/C7 | 48 | 15 (31.3) | 46 | 11 (23.9) | 49 | 11 (22.4) | 48 | 15 (31.3) | |
| Right neural foraminal stenosis C7/T1 | 45 | 0 (0.0) | 46 | 0 (0.0) | 48 | 2 (4.2) | 45 | 0 (0.0) | |
|  | | | | | | | | | |
| Left neural foraminal stenosis C2/C3 | 42 | 1 (2.4) | 44 | 0 (0.0) | 47 | 0 (0.0) | 43 | 0 (0.0) | |
| Left neural foraminal stenosis C3/C4 | 46 | 3 (6.5) | 44 | 0 (0.0) | 49 | 4 (8.2) | 46 | 4 (8.7) | |
| Left neural foraminal stenosis C4/C5 | 48 | 9 (18.8) | 46 | 6 (13.0) | 49 | 6 (12.2) | 48 | 10 (20.8) | |
| Left neural foraminal stenosis C5/C6 | 48 | 13 (27.1) | 46 | 13 (28.3) | 49 | 14 (28.6) | 48 | 16 (33.3) | |
| Left neural foraminal stenosis C6/C7 | 47 | 9 (19.1) | 46 | 8 (17.4) | 49 | 12 (24.5) | 48 | 12 (25.0) | |
| Left neural foraminal stenosis C7/T1 | 45 | 0 (0.0) | 46 | 1 (2.2) | 48 | 0 (0.0) | 45 | 0 (0.0) | |
