# Supplementary material for: Degenerative findings on MRI of the cervical spine: an inter- and intra-rater reliability study
Source: Chiropr Man Therap. 2018 Oct 16;26:43. doi: 10.1186/s12998-018-0210-2 (PMC6190655; doi:10.1186/s12998-018-0210-2)
Supplement: Supplementary file 3 — A table of sensitivity analyses. For neural foraminal stenosis, kappa estimates are presented comparing the assessments of all images vs. only images with available oblique slices. (DOCX 16 kb) [file 12998_2018_210_MOESM3_ESM.docx]

**Sensitivity analyses** Unweighted kappa estimates for foraminal stenosis. Assessment of all images vs. assessment of only images with oblique slices available

| Reader pair | n | All images | n | Only images with oblique slices |
| --- | --- | --- | --- | --- |
| A1B1 | 268 | 0.74 (0.65 ; 0.84) | 232 | 0.74 (0.65 ; 0.84) |
| A1C1 | 287 | 0.73 (0.63 ; 0.82) | 254 | 0.72 (0.62 ; 0.82) |
| B1C1 | 275 | 0.64 (0.53 ; 0.75) | 233 | 0.64 (0.53 ; 0.76) |
| A1B1C1 | 268 | 0.73 (0.63 ; 0.82) | 232 | 0.70 (0.62 ; 0.78) |
